# A novel gene signature unveils three distinct immune-metabolic rewiring patterns conserved across diverse tumor types and associated with outcomes

**DOI:** 10.3389/fimmu.2022.926304

**Published:** 2022-09-02

**Authors:** Leire Pedrosa, Carles Foguet, Helena Oliveres, Iván Archilla, Marta García de Herreros, Adela Rodríguez, Antonio Postigo, Daniel Benítez-Ribas, Jordi Camps, Miriam Cuatrecasas, Antoni Castells, Aleix Prat, Timothy M. Thomson, Joan Maurel, Marta Cascante

**Affiliations:** ^1^ Medical Oncology Department, Hospital Clínic of Barcelona, Translational Genomics and Targeted Therapeutics in Solid Tumors Group, Institut d'Investigacions Biomèdiques August Pi i Sunyer (IDIBAPS), University of Barcelona, Barcelona, Spain; ^2^ Department of Biochemistry and Molecular Biomedicine and Institute of Biomedicine (IBUB), Universitat de Barcelona, Barcelona, Spain; ^3^ Centro de Investigación Biomédica en Red de Enfermedades Hepáticas y Digestivas (CIBEREHD), Instituto de Salud Carlos III (ISCIII), Madrid, Spain; ^4^ Department of Public Health and Primary Care, University of Cambridge, Cambridge, United Kingdom; ^5^ Pathology Department, Hospital Clínic de Barcelona, Barcelona, Spain; ^6^ Group of Transcriptional Regulation of Gene Expression, Institut d'Investigacions Biomèdiques August Pi i Sunyer (IDIBAPS), Institución Catalana de Investigación y Estudios Avanzados (ICREA) and Department of Biomedicine, Universitat de Barcelona, Barcelona, Spain; ^7^ Immunology Department, Hospital Clínic de Barcelona, Barcelona, Spain; ^8^ Gastrointestinal Oncology Department, Hospital Clínic of Barcelona, Institut d'Investigacions Biomèdiques August Pi i Sunyer (IDIBAPS), Barcelona, Spain; ^9^ Department of Cell Biology, Molecular Biology Institute, National Research Council (IBMB-CSIC), Barcelona, Spain; ^10^ Universidad Peruana Cayetano Heredia, Lima, Peru

**Keywords:** biomarker, immunotherapy, precision medicine, metabolism, immune checkpoint-based therapy

## Abstract

Existing immune signatures and tumor mutational burden have only modest predictive capacity for the efficacy of immune check point inhibitors. In this study, we developed an immune-metabolic signature suitable for personalized ICI therapies. A classifier using an immune-metabolic signature (IMMETCOLS) was developed on a training set of 77 metastatic colorectal cancer (mCRC) samples and validated on 4,200 tumors from the TCGA database belonging to 11 types. Here, we reveal that the IMMETCOLS signature classifies tumors into three distinct immune-metabolic clusters. Cluster 1 displays markers of enhanced glycolisis, hexosamine byosinthesis and epithelial-to-mesenchymal transition. On multivariate analysis, cluster 1 tumors were enriched in pro-immune signature but not in immunophenoscore and were associated with the poorest median survival. Its predicted tumor metabolic features suggest an acidic-lactate-rich tumor microenvironment (TME) geared to an immunosuppressive setting, enriched in fibroblasts. Cluster 2 displays features of gluconeogenesis ability, which is needed for glucose-independent survival and preferential use of alternative carbon sources, including glutamine and lipid uptake/β-oxidation. Its metabolic features suggest a hypoxic and hypoglycemic TME, associated with poor tumor-associated antigen presentation. Finally, cluster 3 is highly glycolytic but also has a solid mitochondrial function, with concomitant upregulation of glutamine and essential amino acid transporters and the pentose phosphate pathway leading to glucose exhaustion in the TME and immunosuppression. Together, these findings suggest that the IMMETCOLS signature provides a classifier of tumors from diverse origins, yielding three clusters with distinct immune-metabolic profiles, representing a new predictive tool for patient selection for specific immune-metabolic therapeutic approaches.

## Highlights

- IMMETCOLS signature provides a classifier of tumors from diverse origins.
- IMMETCOLS signature identifies different metabolic patterns associated with immune- suppression.
- IMMETCOLS signature can enable the design of innovative immune-check point- based personalized strategies.

## Introduction

Beyond modulating the nutrient supply to cancer cells or rewiring their metabolism, cellular tumor microenvironment (TME) components can also support neoplastic cells and their growth and dissemination by creating chemical and physical environments that boost tumor-induced immune suppression (1–4) pathways that overlap fitness functions in both cancer cells and TME immune cells. Thus, the transcriptomic profiles of tumor samples recapitulate both cancer cells’ adaptations to nutrient supply and TME-metabolic dependencies. For example, in hypoxic tumors with glucose and glutamine depletion, alternative nutrients such as lipids (5), acetate (6), and extracellular (macropinocytosis) or intracellular (autophagy) bulk proteins (7, 8) can be used to compensate for missing nutrients. In this setting, cells in the TME component like cancer-associated fibroblasts (CAFs) and tumor-associated macrophages (TAMs) can also feed alternative carbon sources, such as nonessential amino acids (9), glutamine (10), or lipids (11, 12) to cancer cells.

A broadly used metric to assess tumor immune microenvironment (TIME) and to predict tumor responsiveness to immune checkpoint immunotherapy (ICI) is immunohistochemistry analysis of programmed cell death ligand (PD-L1) expression. Unfortunately, this metric has been confounded by multiple unresolved issues (e.g., variable detection antibodies, immunohistochemistry cutoffs, tissue preparation, and processing). To improve the predictive accuracy of response to ICI, immunohistochemistry for PD-L1 has been appraised in both tumor epithelial and immune components as part of combined positive scores, with disparate results (13, 14). More recently, gene expression signatures, such as inflammation signatures [T-cell inflammation gene expression profile (GEP) (15), immunophenoscore (IPS) (16), PD-1 expression (17), or tumor mutational burden (18)], have been postulated to improve the predictive accuracy of response to ICI across tumor types. However, the improvement in predictive power afforded by these signatures, by themselves or combined (e.g., GEP and tumor mutational burden), is modest (19). Consequently, we aimed to develop a new immune-metabolic gene signature (IMMETCOLS) that can better identify patients suitable for personalized ICI therapies.

As a starting point, we employed a training set of metastatic colorectal cancer (mCRC) patients yielding three optimal clusters with distinctive immune and metabolic characteristics. The resulting model was subsequently applied to a validation set of 4,200 samples from 11 tumor types, extracted from the TCGA dataset. Based on this classification and the corresponding metabolic features, we propose cluster-specific metabolic targetable vulnerabilities with the potential to synergize with ICI.

## Results

### A new simplified immune-metabolic signature (IMMETCOLS) identifies three subgroups of cancer patients with distinct metabolic and immune-suppressive profiles

**Figure 1:**
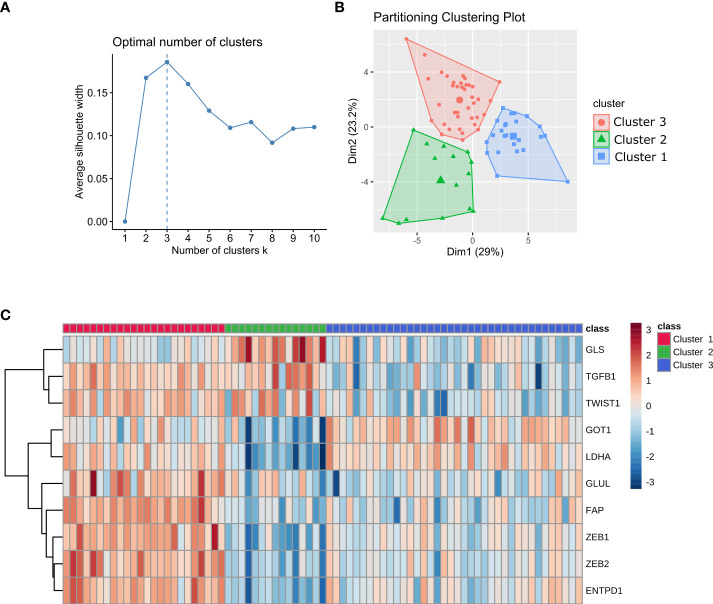
Stratification of mCRC samples into three clusters. **(A)** Plot of average silhouette width vs the number of clusters in k-means. **(B)** Stratification of mCRC samples into three clusters using K-means clustering. **(C)** Heatmap of the gene signature used to classify patients into each cluster. Gene expression values are range-scaled between -3 and 3.

The trained set included 77 mCRC samples. Patient characteristics and initial therapy are presented in **Supplementary Table 1**. Using a custom 770-gene expression panel (IO360) on the NanoString nCounter platform (NanoString Technologies Inc. Seattle, WA, USA), we established three distinct clusters (clusters 1, 2, and 3) (**Figures 1A, B**). Ten genes (*ENTPD1*, *FAP*, *GLS1*, *GLUL*, *GOT1*, *LDHA*, *TGFB1*, *TWIST1*, *ZEB1*, *ZEB2*) were selected as a signature to stratify samples into each cluster (**Figure 1C**). These genes were used as features to train a neural network to classify prospective samples into three clusters. The resulting model yielded 96% accuracy on cross-validation. We have evaluated by immunohistochemistry eight BRAF mutant patients (five patients with high ZEB1 transcriptomic expression and three patients with low ZEB1 transcriptomic expression). Globally, staining was noted in stromal cells but not in epithelial malignant cells (only one patient with high transcriptomic ZEB1 expression showed weak ZEB1 expression in cancer cells). As discussed below, there was no correlation between transcriptomic and immunohistochemistry ZEB1 expression (**Supplementary Figure 1**).

**Figure 2:**
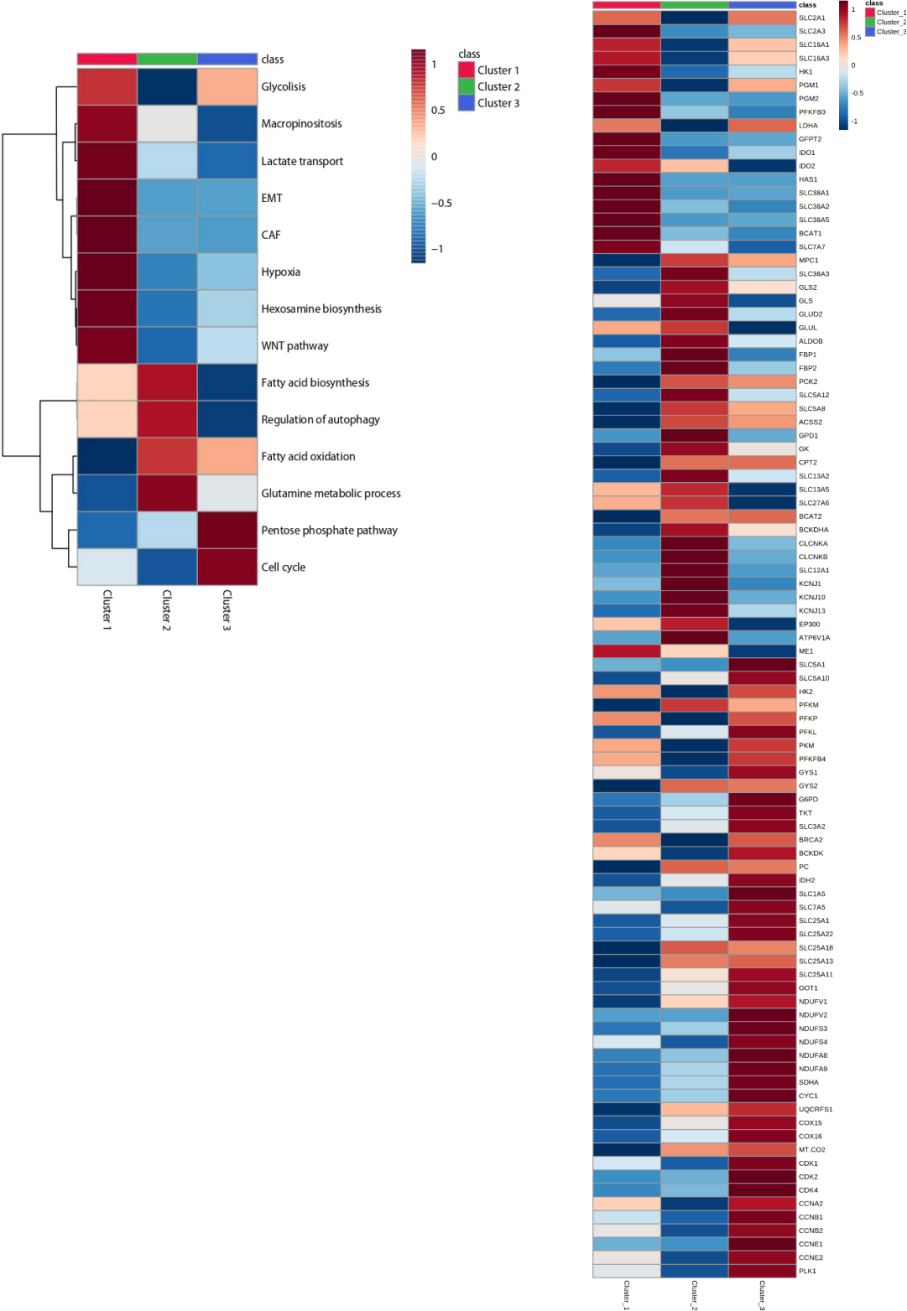
Heatmap of patients classified in Clusters and according to metabolic signatures **(A)** and key enzymes **(B)** expression. The average of gene expression or signature expression in each Cluster is represented in the heatmap. Gene expression values are range-scaled between -1 and 1. On the top or on the left the Cluster classification is shown with red, green or blue, for Cluster 1, Cluster 2 and Cluster 3 respectively.

The resulting trained model was used to stratify 4,200 samples from 11 tumor types in The Cancer Genome Atlas (TCGA) database. Tumor types were selected according to varying clinical response efficacies to pembrolizumab into three groups: sensitive, with the best-observed response (BOR) between 28% and 36% (SKCM, LUAD, KIRC, and BLCA); moderately sensitive, with BOR between 7% and 17% (BRCA-TNB, OV, HNSC, STAD); and resistant, with BOR between 0% and 8% (COAD-MSS, PAAD, GBM). The three identified clusters display distinct immune and metabolic characteristics with significant differences in the expression levels of transcripts for metabolic enzymes and immune markers that define distinct immune and metabolic characteristics for each cluster (**Figures 2A, B** **;** **Supplementary Figures 2, 3** **;** **Supplementary Tables S2–S4**). We provide below a detailed description of the main metabolic characteristics inferred for each of these three clusters.

#### Cluster 1: Mesenchymal glycolytic

About 20% to 36% of the cases were classified into cluster 1. Tumors in this cluster display a differential upregulation of glucose transporters, glycolytic enzymes, and lactate transporters (including SLC16A1, which also transports branched-chain ketoacids), strongly suggestive of an efficient use of glucose and glycolysis as the major source of carbon, energy, and downstream metabolites. The inferred funneling of glycolysis toward the production and efflux of lactate, concomitant with the diminished expression of key players in mitochondrial pyruvate transport (MPC1) and oxidation [i.e., isocitrate dehydrogenase isoenzymes (IDH) and respiratory mitochondrial complexes] (**Figures 2A, B** **;** **Supplementary Figure 3**), suggests a reduced activity of the oxidative tricarboxylic acid (TCA) cycle and mitochondrial respiration [oxidative phosphorylation (OXPHOS)] consistent with a Warburg effect taking place in the tumors in this cluster (20–22). Cluster 1 tumors are also enriched in hypoxia-induced glutamine transporters, including glutamine/H^+^ antiporter that leads to cell alkalinization through the removal of H^+^. Indeed, cluster 1 is also enriched for the expression of BCAT1, involved in deamination/transamination of keto/amino acids, and for the expression of enzymes in the hexosamine and hyaluronan biosynthetic pathways, predicting an enhanced synthesis of uridine diphosphate N-acetylglucosamine. Another salient inferred metabolic feature in this cluster is the overexpression of tryptophan-degrading enzymes, indoleamine-2,3-dioxygenase-1 (IDO1) and tryptophan-2,3-dioxygenase (TDO2), that results in the production of the powerful immunosuppressant metabolite kynurenine and of SLC7A7 and WARS1 (tryptophanyl-tRNA synthetase) (23) (**Figure 2B**, **Supplementary Figure 4**, **Supplementary Table 3**). From the above inferences, the metabolic scenario for cluster 1 is that of tumors geared for glucose consumption and, even more prominently, optimally adapted to acidic, glutamine-, and tryptophan-starved TME.

#### Cluster 2: Epithelial non-glycolytic

The most prominent metabolic inferred feature of tumors in cluster 2 (13% to 19% of the cases) is the positive enrichment of key enzymes of gluconeogenesis, concomitant with increased expression of pathways for the use of carbon sources other than glucose and diminished expression of key players in glycolysis and glucose transport. The enrichment in gluconeogenesis is strongly supported by the upregulation of genes coding for phosphoenolpyruvate carboxykinase (PKC2), aldolase B (ALDOB), and fructose-1,6-bisphosphatase isoenzymes (FBP1 and FBP2) (see **Figures 2A, B**, **Supplementary Figure 4**).

On one hand, this is compensated by the upregulation of components of glutamine transport [SLC38A3, glutaminase (GLS1 and GLS2), and glutamate dehydrogenase (GLUD2)]. Interestingly, cluster 2 tumors display also proper adaptation to a glutamine-deprived microenvironment through upregulated glutamate-ammonia ligase (GLUL) and upregulated branched-chain amino acid aminotransferase (BCAT2). On the other hand, cluster 2 tumors also display upregulation of the BCKDHA component of the BCK complex, the rate-limiting step in the branched-chain amino acid (BCAA) catabolic pathway, resulting in enhanced synthesis of the end products succinyl-CoA and acetyl-CoA. The gluconeogenesis pathway is further reinforced in cluster 2 by the upregulation of glycerol kinase (GK) and glycerol-3-phosphate dehydrogenase (GPD1). It is worthy to note that histone acetyltransferase p300 (EP300) is also overexpressed in cluster 2 (see **Figure 2B**, **Supplementary Figure 4**) (4).

Other carbon sources enriched in cluster 2 tumors are long-chain fatty acids (LCFAs), transported by SLC27A6 (FATP6), and short-chain fatty acids (SCFAs), acetate, ketone bodies, lactate, and other gluconeogenic precursors, transported by SLC5A8 and SLC5A12. The observed enrichment on acetyl-CoA synthetase (ACSS2) ensures the production of cytosolic acetyl-CoA from the imported acetate and the upregulation of CPT2 and facilitates the translocation of LCFAs into the mitochondria as a fuel to feed β-oxidation for ATP production. SLC13A2 and SLC13A5 are also upregulated supporting an enhanced import of citrate into cancer cells in cluster 2 tumors (**Figure 2B**, **Supplementary Figure 4**). Our observations are consistent with the increasing evidence that macrophages secrete citrate and fatty acids (11, 24) and thus “feed” cancer cells (25, 26) (see **Figure 5**).

**Figure 3:**
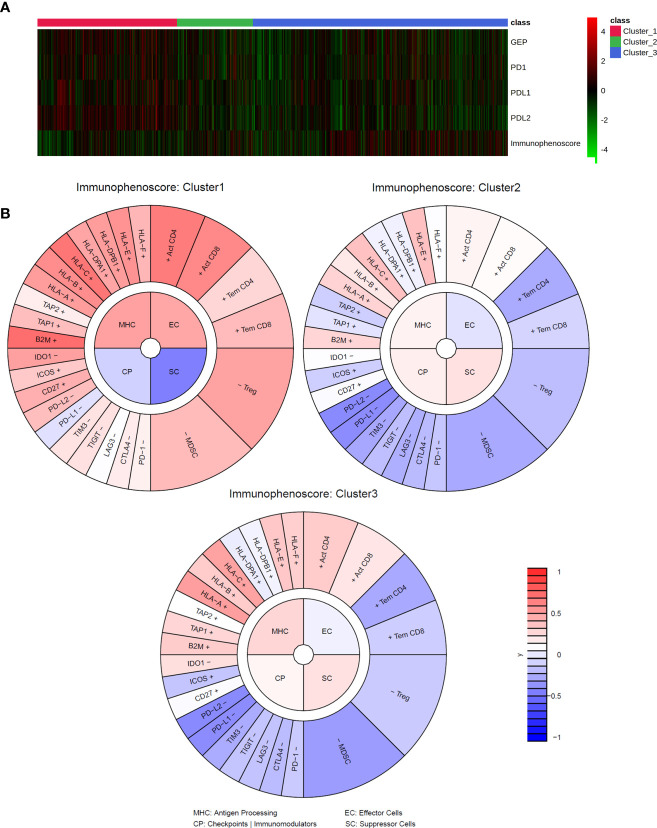
Immune signatures and IMMETCOLS. **(A)** heatmap of transcriptomics immune signatures in TCGA samples stratified according to IMMETCOLS. GEP is the average expression of the genes of the GEP signature. Immunophenoscore is the aggregate of the MHC (Antigen Processing), EC (Effector cells), CP (Checkpoints and Immunomodulators) and SC (Suppressor cells) scores. **(B)** Average immunophenogram in each IMMETCOLS cluster. Inner circle plots each of the four Immunophenoscore components with higher values representing a more immunogenic phenotype. The outer cycle plots the expression of markers used to compute each of the immunophenoscore components.

Cluster 2 tumors are also enriched in v-ATPase (ATP6V1A), the key proton pump for endolysosomal acidification, and on the ion transporters SLC12A1 (NKCC1), CLCNKA, CLCNKB, KCNJ1, KCNJ10, and KCNJ13, with genes implicated in the canonical autophagy pathway (see **Figure 2B**, **Supplementary Figure 3**). In accordance with our results, enhanced autophagy/lysosome function has been described to result in MHC-I degradation and immune evasion (27, 28) (see **Figure 3B**).

#### Cluster 3: Epithelial glycolytic oxidative mitochondrial

Cluster 3 is the most prevalent category in the overall set of tumors and for all the tumor types analyzed (49% to 63% of the cases). Tumors in this cluster are enriched for genes for many components of the electron transport chain: complex I (NDUFVs, NDUFSs, NDUFAs, NDUFBs, NDUFCs, NDUFAF1, NDUFS4), complex II (SDH), complex III (CYC1, UQCRs), and complex IV (COXs, MT-CO2) (**Figure 2B**, **Supplementary Figures 4, 5**). Moreover, several mitochondrial carriers of the SLC25 family and the malate–aspartate shuttle are also overexpressed. In accordance with the upregulation of the malate–aspartate shuttle, upregulation of aspartate aminotransferases (GOT1) has been also observed. Moreover, the observed enrichment on glutamine and BCAA transporters (SLC1A5 and SLC7A5) and on the BCAA catabolism pathway (SLC3A2, BCA2, and BCKDK) strongly supports the use of these metabolites as predominant sources of carbons feeding mitochondrial activity in cluster 3 tumors. Notably, key players in conferring capacity to perform reductive carboxylation, from a-ketoglutarate to citrate, such as pyruvate carboxylase (PC), isocitrate dehydrogenase (IDH2), and key enzymes in proline biosynthesis, and one carbon and folate metabolism are also overexpressed and generate a need for glutamine and NADPH to sustain these pathways (**Figure 2B**, **Supplementary Figure 4**). Finally, cluster 3 is characterized by the overexpression of GLUT1 (SLC2A1) and sodium-dependent glucose cotransporters (SLC5A1 and SLC5A10), glycolytic enzymes (HK2, PFKl, PFKP,PKM1, and PFKFB4), glycogen synthase isoenzymes (GYSs), and key players in pentose phosphate pathways (PPPs) (G6PD and TK) and diminished expression of cytosolic malic enzyme (ME1) (**Figure 2B**, **Supplementary Figure 4**). The observed overexpression of Polo-like kinase 1 (PLK1), a direct activator of G6PD (3), strongly supports the cluster 3 tumors’ dependence on PPPs. The enrichment of key players of the OXPHOS and PPPs confers to cluster 3 tumors an enhanced capacity to sustain cell division. Indeed, a supervised examination of genes for cell cycle regulators indicates a preferential enrichment of cell cycle driver genes (CDK1-2 CDK4 and cyclins CCNA2, CCNB1-2, and CCNE1-2).

### Association of IMMETCOLS metabolic subtypes with pro-immune signatures

To explore the usefulness of the IMMETCOLS classification in the prediction of tumor immune microenvironments across tumor types, we explored the TCGA database (*n* = 4,200 cases) for relationships between IMMETCOLS and several proinflammatory signatures, including the T-cell-inflamed gene expression profile (GEP, encompassing genes related to cytolytic activity, inflammatory cytokines/chemokines, T and NK cell markers, antigen presentation, and other immunomodulatory factors) and the expression of CD274 (PD-L1), PD-L2, PD-1, or immunophenoscore [scoring MHC expression, effector cells (ECs), checkpoint immunomodulators (CPs), and suppressor cells (SCs)] (**Supplementary Figure 2**, **Supplementary Tables 4, 5**).

To this end, cases stratified by GEP, PD-1, PD-L1, and PD-L2 and immunophenoscore were assessed for enrichment in the IMMETCOLS signature. We found that the GEP signature [false discovery rate (FDR) = 1.91 × 10^–08^] and the expression levels of PD-L1 (FDR = 5.16 × 10^−06^), PD-L2 (FDR = 6.12 × 10^−19^), and PD-1 (FDR = 9.204 × 10^−4^) were upregulated in cluster 1 IMMETCOLS samples (**Figure 3A**). In contrast, the immunophenoscore signature was upregulated in clusters 2 and 3 IMMETCOLS samples (FDR = 2.12 × 10^−4^) (**Figure 3B**). Of note, cluster 1, despite showing the highest enrichment in MHC and CD8, had the lowest immunophenoscore, attributable to the upregulation in CP (FDR = 1.72E−12) and SC (FDR = 1.72E−25) (**Figure 3B**). A similar pattern was observed in the training set of mCRC patients (**Supplementary Figure 5**), where PD-L1 (FDR = 2.44 × 10^−4^), PD-L2 (FDR = 8.5510−13), PD-1 (FDR = 5.95 × 10^−3^), and the GEP signature (FDR = 6.26 × 10^−4^) were upregulated in cluster 1. Likewise, in the set of mCRC patients, immunophenoscore was significantly lower in cluster 1 than in clusters 2 and 3 (FDR = 7.75 × 10^−5^) driven by an increased SC signature (FDR = 9.6 × 10^−7^). Finally, as illustrated in **Supplementary Figure 6**, tumors refractory to pembrolizumab therapy (COAD-MSS, PAAD, GBM) with high GEP scores or PD-1 expression fall almost entirely within the immunosuppressive cluster 1.

### Relevance of the IMMETCOLS signature as a prognostic score

**Figure 4:**
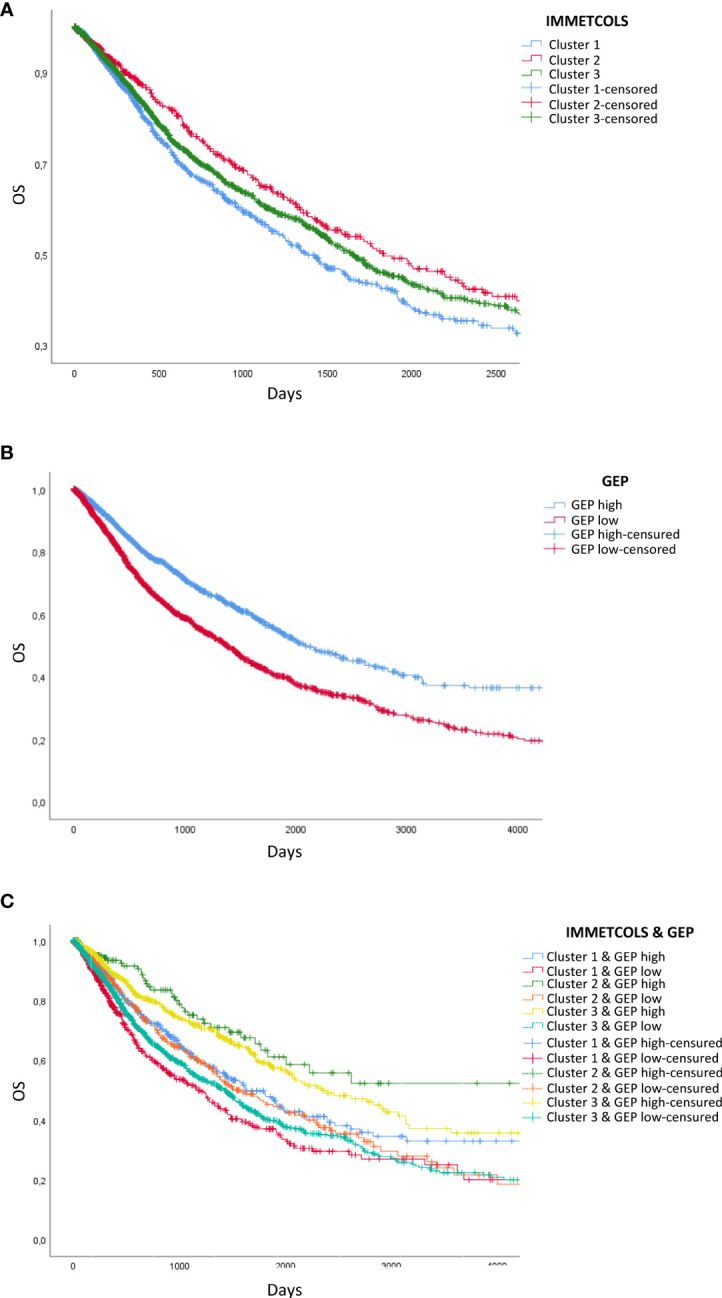
Survival analysis of TCGA patients classified by GEP- and IMMETCOLS Signature expression. **(A)** Overall survival of patients classified by IMMETCOLS signature. **(B)** Kaplan Meier Curves compare patients with high GEP expression versus patients with low GEP expression. **(C)** Overall survival of patients classified according to GEP and IMMETCOLS expression.

Given the observed association between TIME classifiers and the three IMMETCOLS clusters and prior evidence that the T-cell-inflamed GEP and PD-1 expression levels are predictors of clinical response to ICI, we assessed the prognostic value of these signatures (GEP, PD-1, and IMMETCOLS) in the 11 tumor types. As expected, patients with high GEP and high PD-1 presented a longer overall survival (**Figure 4B**, **Supplementary Figure 7A**). In contrast, patients with tumors enriched in the IMMETCOLS cluster 1 signature exhibited poor survival in the univariate analysis (**Figure 4A**). This is at apparent odds with the correlation found above between IMMETCOLS cluster 1 and the immune signatures. This discrepancy could potentially reflect the heterogeneous response patterns to ICI therapy in the tumor groups stratified by each or all of these classifiers.

As such, we extended our analysis by considering the combinations of GEP, PD-1, and IMMETCOLS signatures as potential predictors of subgroups of cases with diverse prognoses. We found that while patients with high GEP scores and enriched in IMMETCOLS cluster 2 or 3 signatures had the most favorable OS, patients with low GEP scores and enriched in IMMETCOLS cluster 1 signature had the poorest OS (**Figure 4C**, **Supplementary Figure 7B**). At multivariate analysis, age, tumor type, and IMMETCOLS signature remained significant (**Table 1**).

**Table 1:** Univariate and multivariate analysis with demographic variables, PD1 expression, type of tumour, MHC expresion and by GEP and IMMETCOLS classification.

Univariate				
			95% CI HR	
	Sig.	HR	Inferior	Superior
**IMMETCOLS**	0,028			
**Gender**	0,829	1,012	0,909	1,127
**Age at initial diagnosis**	0,000	1,023	1,019	1,028
**GEP**	0,000	0,809	0,763	0,859
**PD1**	0,000	0,822	0,770	0,878
**MHC**	0,000	0,741	0,675	0,813
**Type of tumour**	0,000			

Mutivariate				
			95% CI HR	
	Sig.	HR	Inferior	Superior
**IMMETCOLS**	0,022			
**Gender**	0,407	0,948	0,835	1,076
**Age at initial diagnosis**	0,000	1,024	1,019	1,029
**GEP**	0,079	0,846	0,701	1,020
**PD1**	0,714	1,096	0,671	1,789
**MHC**	0,270	0,922	0,798	1,065
**Type of tumour**	0,000			

## Discussion

**Figure 5:**
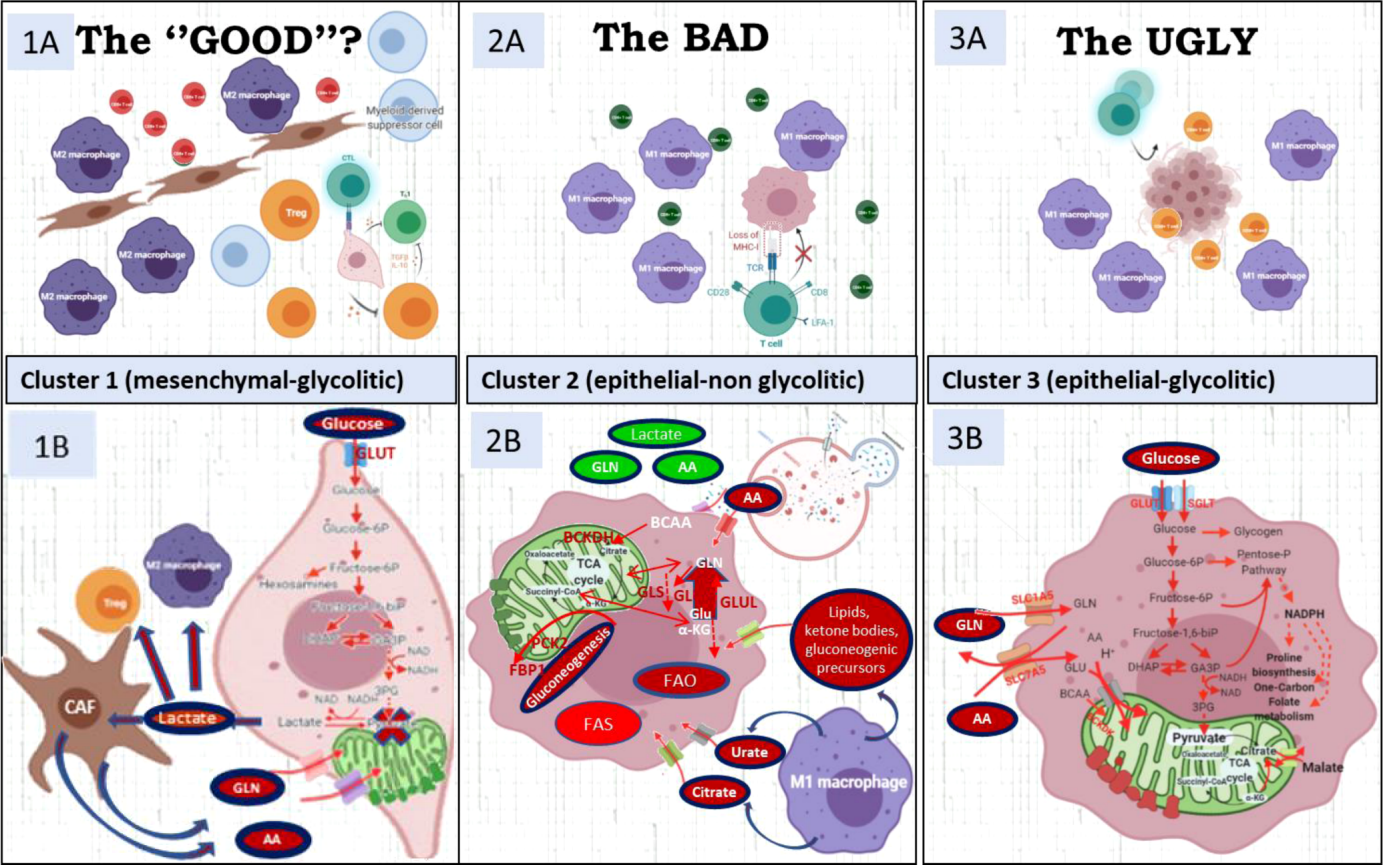
The upper file describes the micro-environment characteristics of the three clusters. The lower file describes the metabolic characteristics of each cluster. **(1A)** Highly immune-suppressive microenvironment with M2 polarized TAM and Tregs characterizes cluster 1. CD39, IDO-1, TGFb1, CD47, IL-10, contribute to immunosuppression. **(1B)** Cancer mesenchymal cells use pyruvate to produce lactate to feed CAF and these CAFs sustain with alternative carbon and nitrogen sources the TCA cycle in cancer cells. **(2A)** CD8 have bystander characteristics (CD39-, PD1-) due to cancer-cell MCH-I low presentation. **(2B)** Cluster 2 has enhanced glutamine/BCKA oxidation and gain of gluconeogenic/glycogenic ability which are needed for glucose-independent survival and up-regulated enzymes in lipids b-oxidation and glutamine synthesis. **(3A)** CD8/PD1+ and M1 macrophages compete with cancer cells for glucose and glutamine **(3B)** Its metabolic signature includes up-regulation of key enzymes in proline synthesis, one-carbon metabolism and key players of the malate-aspartate shuttle, suggestive of a gain of reductive carboxylation ability. Figure created with BioRender.com.

Given the inferred high glucose, glutamine, and tryptophan depletion of the TME due to consumption by cancer cells in cluster 1 tumors, in concert with high lactate secretion, the predicted overall scenario is that of an immunosuppressive setting, rich in interferon-γ-producing cells surrounding neoplastic cells (**Supplementary Figure 2**). In fact, IFNγ also overexpressed in cluster 1, induces WARS and tryptophan catabolism enzymes (29). A further prediction is that CAFs likely recruited to the tumor site by a cytokine-rich environment may use tumor-produced lactate as a source of carbon and, in return, release to the TME further metabolites such as glutamine and other amino/keto acids that contribute to tumor growth and reinforcement of an immunosuppressive tumor microenvironment (4) (**Figure 5**). These predictions are supported by evidence for a CAF-populated and a highly suppressive TIME in cluster 1 tumors, with enrichment for markers for regulatory T cells (Tregs) (2, 30), macrophages with M2 polarization (1, 31, 32), myeloid-derived suppressor cells (MDSCs), and exhausted CD39^+^CD8^+^ T cells (33, 34). Because many immunosuppressive mechanisms coexist in this cluster (CD47, LAG-3, IDO-1, TGFB, CD39/CD73) between others (**Figure 3B**, **Supplementary Figure 2**, **Supplementary Tables 3, 4**), it is unlikely that ICI combined with current drugs in clinical development blocking these targets (magrolimab, relatlimab or favezelimab, epacadostat/BMS-986205, NIS793, SRF617/NZV930, respectively) would be able to increase significantly the efficacy of current therapies.

The glucose, tryptophan, and glutamine dependencies inferred for cluster 1 tumors support the use of inhibitors of key enzymes and transporters in the corresponding pathways as strategies to undermine the fitness of neoplastic cells while countering the immunosuppressive environment. Inhibitors of glucose import or rate-limiting glycolytic enzymes are predicted to have an impact on tumor cells and also on the acidification of the TME by mitigating the production and secretion of lactate, with consequent relief from an immunosuppressive TME. Lactate transport inhibitors [e.g., AZD3965 or diclofenac (35)], predicted to have an impact by resulting in intracellular lactate accumulation and increased synthesis of ROS, and/or hexosamine biosynthesis inhibitors, such as 6-diazo-5-oxo-L-norleucine (36) that is expected to impair the stabilization of PD-L1 through glycosylation and favoring infiltration of CD8^+^ T cells through decreasing hyaluronan synthesis for the ECM, could increase the efficacy of ICI therapy in this cluster.

The existence of a highly stressful environment in cluster 2 tumors, with nutrient scarcity and low oxygen tension, is reinforced by the observed reduced MHC-I expression and bystander CD39^+^CD8^+^ cells (37). We hypothesize that autophagy inhibitors such as chloroquine (27) or selective autophagy target inhibitors (38) such as metformin (a citrate transport and gluconeogenesis inhibitor) (39, 40), or CCS1477 (an inhibitor of p300/CBP) currently in clinical development (41, 42), can increase ICI efficacy in this cluster.

The enrichment of genes and pathways in cluster 3 predicts an enhanced metabolic plasticity to adapt to low-/high-oxygen levels and a stressful nutritional environment. Thus, overexpression of key pathways supporting the reductive carboxylation pathway (aKG conversion to citrate through IDH2) facilitates glutamine conversion to citrate in hypoxic conditions, and overexpression of OXPHOS genes ensures an increased ATP production capacity in an oxygen-abundant environment. Given this scenario, cluster 3 tumors are predicted to be sensitive to cytotoxic drugs that target mitochondrial metabolism, glutamine and amino acid intake and metabolism (e.g., the GLS inhibitor CB-839), amino acid transport inhibitors (43), glucose transport inhibitors (44), and the CDK inhibitors (e.g., palbociclib or dinaciclib). However, although CDK inhibitors could inflame cold tumors (45), we recommend caution with this strategy because they can stimulate oxidative stress in cancer cells (46, 47) due to glucose, glutamine, and lipid competition with immune cells (48–50) and increase the antioxidant network (51).

Given the toxicity induced by the concomitant use of abemaciclib and pembrolizumab (52), we propose a sequential strategy with CDK4/6 inhibitors followed by ICI and drugs that inhibit antioxidant pathways (e.g., onvansertib, a PLK1 inhibitor).

To improve the clinical utility of biomarker ICI prediction, we evaluated our IMMETCOLS signature with previously published pro-immune signatures (GEP, PD-1, PD-L1) and immunophenoscore. We hypothesize that capturing both pro-immune and IMMETCOLS signatures could potentially allow better ICI accuracy than the signatures separately. Although cluster 1 is enriched with pro-immune signatures, it does not translate to a higher immunophenoscore. This issue has been described particularly in tumors with a signature enriched with transforming growth factor β and EMT features that usually show an immuno-excluded phenotype when inflamed (53, 54). Although we evaluate also the transcriptomic protein correlation of *ZEB1* (a well-known EMT gene) in selected BRAF mutant samples, we do not find a correlation. Although the number of analyzed cases is limited, these results emphasize the difficulty particularly for genes like *ZEB1* whose expression in tumor cells is limited to a few scattered cells. Finally, cluster 1 (mesenchymal subtype) showed the worst prognosis in accordance with previous publications (55), and despite this, we have to note that survival differences in the TCGA dataset are curtailed by the scarcity of clinical data in that platform, possibly affecting the multivariate analysis.

In cluster 1, which shows a mesenchymal phenotype with glycolytic Warburg metabolism and lactate export, the MCT1 inhibitor (AZD3965) in combination with ICI increases ICI activity, reducing tumor efflux of lactate (35) and decreasing lactate uptake by Tregs (56, 57). In clusters 2 and 3 that rely on OXPHOS, OXPHOS inhibitors would increase ICI sensibility in melanoma patients that progressed with PD-1 and CTLA4 blockade (58, 59) and in melanoma brain metastases that rely on OXPHOS (60).

In conclusion, this study provides evidence that the IMMETCOLS signature can identify patient subsets from diverse tumor types for optimally tailored ICI therapy in combination with metabolic modulators to potentiate antitumor immunity reactivation. Patient stratification should be readily amenable through the 10-gene NanoString platform described here.

## Methods

### Patient cohort

The use of human samples was approved by the Clinical Ethics Research Committee at the Hospital Clinic of Barcelona (references HCB-2013/8674 and HCB-2018/0633). This study includes a retrospective cohort of 128 patients diagnosed with mCRC at the HCB. Patients signed an informed consent approving the ulterior use of their tumor specimens in the GEMCAD collection repository.

### RNA extraction and NanoString gene expression profiling

The NanoString IO360 panel on the nCounter platform (NanoString Technologies) was used to interrogate gene expression on FFPE tissue following the manufacturer’s protocol. Briefly, 10-µm-thick sections of formalin-fixed paraffin-embedded (FFPE) tumor tissues were examined by H&E. Only samples with ≥10% tumor cellularity were processed for RNA purification (High Pure FFPET RNA isolation kit, Roche, Roche Diagnostics Limited, West Sussex, UK). When needed, macrodissection was performed to enrich tumor cells and minimize stromal components. After excluding samples with suboptimal RNA integrity and content, the remaining samples were included in the nCounter analysis. The final set of data (*n* = 77) was analyzed on the nSolver 4.0 Advanced Analysis module using default settings to derive differentially expressed genes, pathway scores, and cell type scores.

### Immunohistochemistry

Formalin-fixed paraffin-embedded 2-μm-thick sections were used for ZEB1 immunohistochemistry. After standard antigen retrieval with buffer pH 6.1 (Dako, Agilent, Santa Clara, CA, USA), the ZEB1 antibody (polyclonal Atlas Antibody. Ref HPA027524) was incubated for 30 min following the standard immunohistochemistry protocol, developed with DAB. Normal rabbit IgG isotypes were applied as a negative control (data not shown). Immunohistochemical staining was independently evaluated by two gastrointestinal pathologists blinded to any other information using an optical microscope Olympus BX41 (Olympus Corporation, Tokyo, Japan). The evaluation of the intensity of the immunostains was performed using a semiquantitative grading system into absent, low, or intense expression in both epithelial and stromal cells. The pattern of immunostaining was nuclear or cytoplasmic.

### Clustering and neural network analysis for tumor classification

NanoString (IO360) expression data from 77 mCRC samples were mean-centered and scaled by subtracting the mean and dividing by the standard deviation of each gene across all samples. The average silhouette method was applied to establish optimal cluster numbers. A one-way ANOVA of gene expression with cluster as a factor was used to identify 10 differentially expressed genes that could be used to separate the three clusters. These genes were used as features to train a neural network to classify prospective samples into the three clusters. To this end, the “nnet” implementation of the R Caret package was used. A 10-fold cross-validation with 100 iterations was performed on the training data to fit the two hyperparameters of the neural network (size and decay).

Gene counts for individual patients in the TCGA were normalized using the variable stabilizing transformation (VST) function of the DESeq2 package for R. The expression levels for each gene in the 10-gene signature were mean-centered and scaled in each tumor type and used as input of the Caret predict function to stratify samples into our clusters. ANOVA was used to compare gene expression and immune signatures between samples assigned to each cluster. *Post-hoc* analyses to assess level differences were performed with Fisher’s least significant difference method (Fisher’s LSD).

### Immune signatures

Samples were assigned to GEP-High if the average expression of genes of the GEP signature was above the 66th percentile of the average expression of GEP genes in all analyzed tumors (15). Samples were considered to be PD-1-High or PD-L1-High if the expression of such markers was above the 75th percentile in all analyzed tumors. Immunophenogram and immunophenoscore components and markers were evaluated and plotted using the function developed by Charoentong et al. (16).

### Other statistical methods

Log-rank analysis was performed to determine the statistical significance of the Kaplan–Meier survival curves using SPSS v.25 (IBM, Armonk, NY, USA) software. The time-to-event point was considered for survival analysis: the primary endpoint was overall survival (OS), defined as the time from metastatic diagnosis to death from any causes (for deceased patients). For univariate and multivariate analyses, descriptive statistics were used to test their correlation with overall survival (OS).

## Data availability statement

The data presented in the study are deposited in the GEO repository, accession number GSE206613. https://www.ncbi.nlm.nih.gov/geo/query/acc.cgi?acc=GSE206613

## Ethics statement

The studies involving human participants were reviewed and approved by Ethics Committee of the Hospital Clinic of Barcelona. The patients/participants provided their written informed consent to participate in this study.

## Author contributions

Conceptualization: TT, JM, and MCa. Data curation: LP, CF, JM, and MCa. Funding acquisition: APo, TT, JM, and MCa. Investigation: LP, CF, MCu, JM, and MCa. Resources: JM and MCa. Supervision: JM and MCa. Writing—review and editing: LP, CF, HO, IA, MG, AR, APo, DB-R, JC, MCu, AC, AP, TT, JM, and MCa. All authors have read and agreed to the published version of the manuscript.

## Funding

This study was supported by grants from the Spanish Ministry of Science and Innovation (MICINN) SAF2017-84918-R0 (National Scientific and Technical Research and Innovation 2017–2020 Plan, co-funded by the EUC-ERDF) to APr, PID2019-107139RB-C21 to TT, and PID2020-115051RB-I00 to MCa funded by MCIN/AEI/10.13039/501100011033; grants from Fundació la Marató de TV3 (201330.10) to APo, Foundation Olga Torres (Biannual Grant A-2019/2020) to APo, and Fundació ICREA-Premi Icrea Academia to MCa; grants from Catalan Agency for Management of University and Research Grants (AGAUR) 2017-SGR-1174 to APo and 2017-SGR-1033 to MCa and Spanish Association Against Cancer (AECC, PROYE19040POST_001) to APo and JM; and grants from Instituto de Salud Carlos III (PI13/01728 and PI19/0740) to JM.

## Conflict of interest

JM received research grants from Merck, Roche, Amgen, NanoString, Incyte, and Biocartis and reports personal fees from Advance Medical, Cancer Expert Now, Fundación Clínica Universitaria, Sirtex, Pierre-Fabre, Shire, AstraZeneca, Bayer, Servier, Sanofi, and Roche.

The remaining authors declare that the research was conducted in the absence of any commercial or financial relationships that could be construed as a potential conflict of interest.

## Publisher’s note

All claims expressed in this article are solely those of the authors and do not necessarily represent those of their affiliated organizations, or those of the publisher, the editors and the reviewers. Any product that may be evaluated in this article, or claim that may be made by its manufacturer, is not guaranteed or endorsed by the publisher.
