# Supplementary material for: A novel gene signature unveils three distinct immune-metabolic rewiring patterns conserved across diverse tumor types and associated with outcomes
Source: Front Immunol. 2022 Sep 2;13:926304. doi: 10.3389/fimmu.2022.926304 (PMC9479210; doi:10.3389/fimmu.2022.926304)
Supplement: Supplementary file 1 [file DataSheet_1.docx]

Supplementary Figure S1: ZEB1 immunohistochemistry (IHC) showed different expression patterns, with stromal cells with intense positivity and absent expression in tumoral cells (A), the weak expression on epithelial cells intensity (B) and stromal cells (C) and absent reactivity in both epithelial and stromal components (D) (x200). Solid arrow: Positive stromal cells. Dotted arrow: Positive epithelial cells.
