# Supplementary material for: A novel gene signature unveils three distinct immune-metabolic rewiring patterns conserved across diverse tumor types and associated with outcomes
Source: Front Immunol. 2022 Sep 2;13:926304. doi: 10.3389/fimmu.2022.926304 (PMC9479210; doi:10.3389/fimmu.2022.926304)
Supplement: Supplementary file 2 [file DataSheet_2.docx]

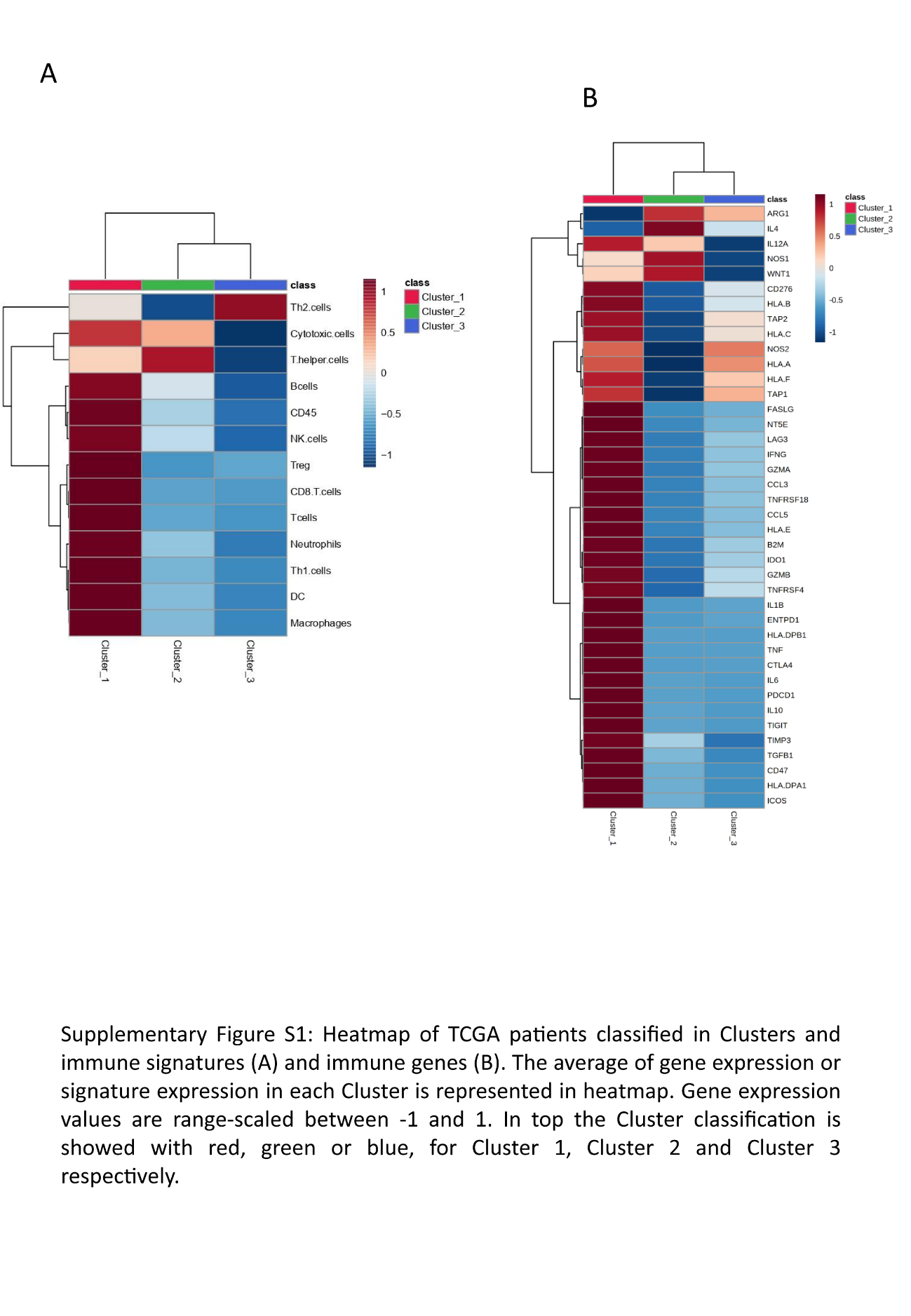


Supplementary Figure S2: Heatmap of TCGA patients classified in Clusters and immune signatures (A) and immune genes (B). The average of gene expression or signature expression in each Cluster is represented in heatmap. Gene expression values are range-scaled between -1 and 1. In top the Cluster classification is showed with red, green or blue, for Cluster 1, Cluster 2 and Cluster 3 respectively.
