# Supplementary material for: A novel gene signature unveils three distinct immune-metabolic rewiring patterns conserved across diverse tumor types and associated with outcomes
Source: Front Immunol. 2022 Sep 2;13:926304. doi: 10.3389/fimmu.2022.926304 (PMC9479210; doi:10.3389/fimmu.2022.926304)
Supplement: Supplementary file 3 [file DataSheet_3.docx]

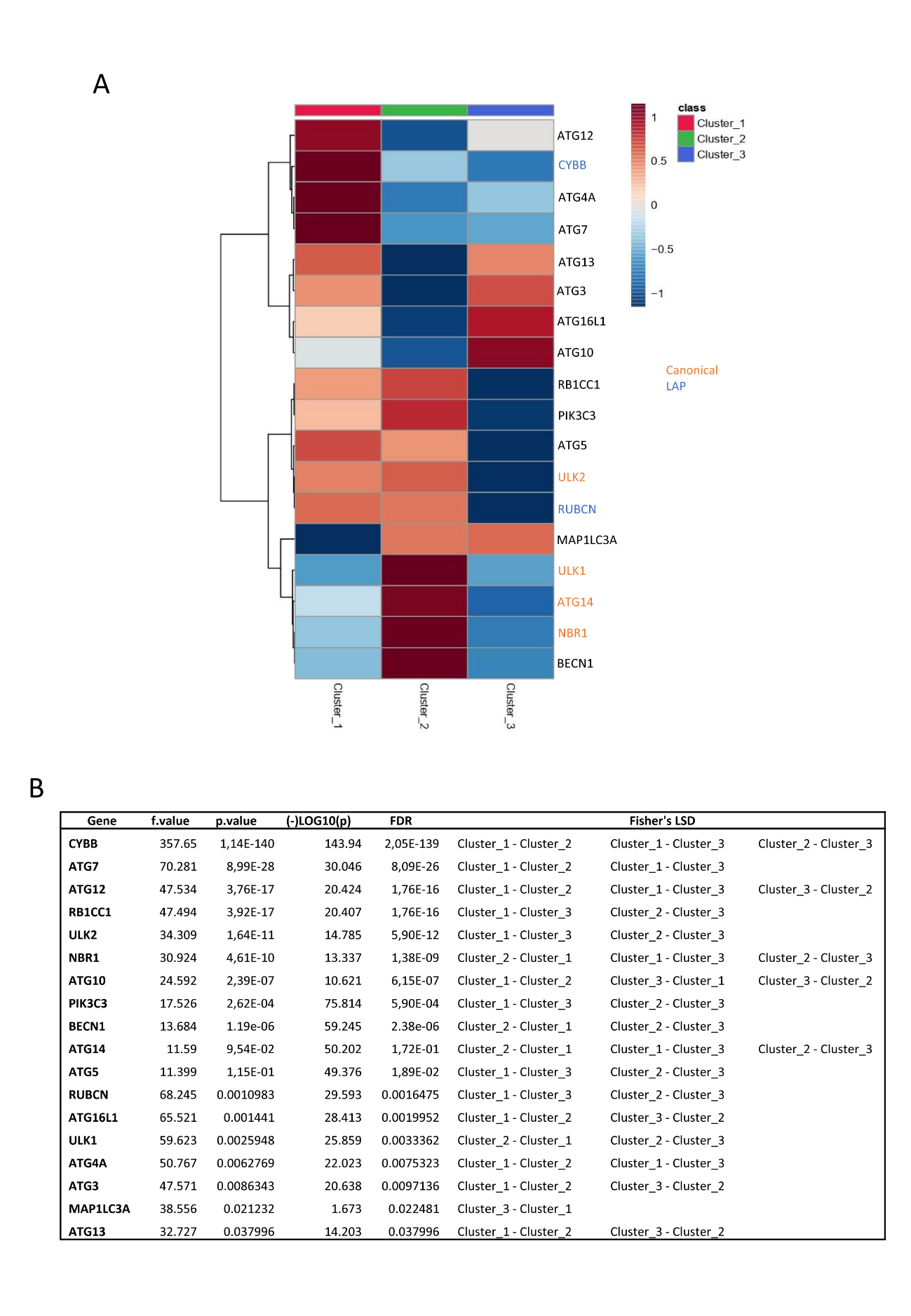


Supplementary Figure S3: Heatmap (A) and statistic results (B) studding the expression of genes related with autophagy in IMMETCOLS Clusters. (A) Heatmap representing the average expression of genes related to autophagy in IMMETCOLS Cluster. Gene expression values are range-scaled between -1 and 1. In top the Cluster classification is showed with red, green or blue, for Cluster 1, Cluster 2 and Cluster 3 respectively. The genes of canonical autophagy are in orange circle and the LAP genes are in blue circles. (B) Important features identified by One-way ANOVA and post-hoc analysis (Fisher’s LSD) comparing the expression of genes related to autophagy in the IMMETCOLS Clusters.
