# Supplementary material for: A novel gene signature unveils three distinct immune-metabolic rewiring patterns conserved across diverse tumor types and associated with outcomes
Source: Front Immunol. 2022 Sep 2;13:926304. doi: 10.3389/fimmu.2022.926304 (PMC9479210; doi:10.3389/fimmu.2022.926304)
Supplement: Supplementary file 4 [file DataSheet_4.docx]

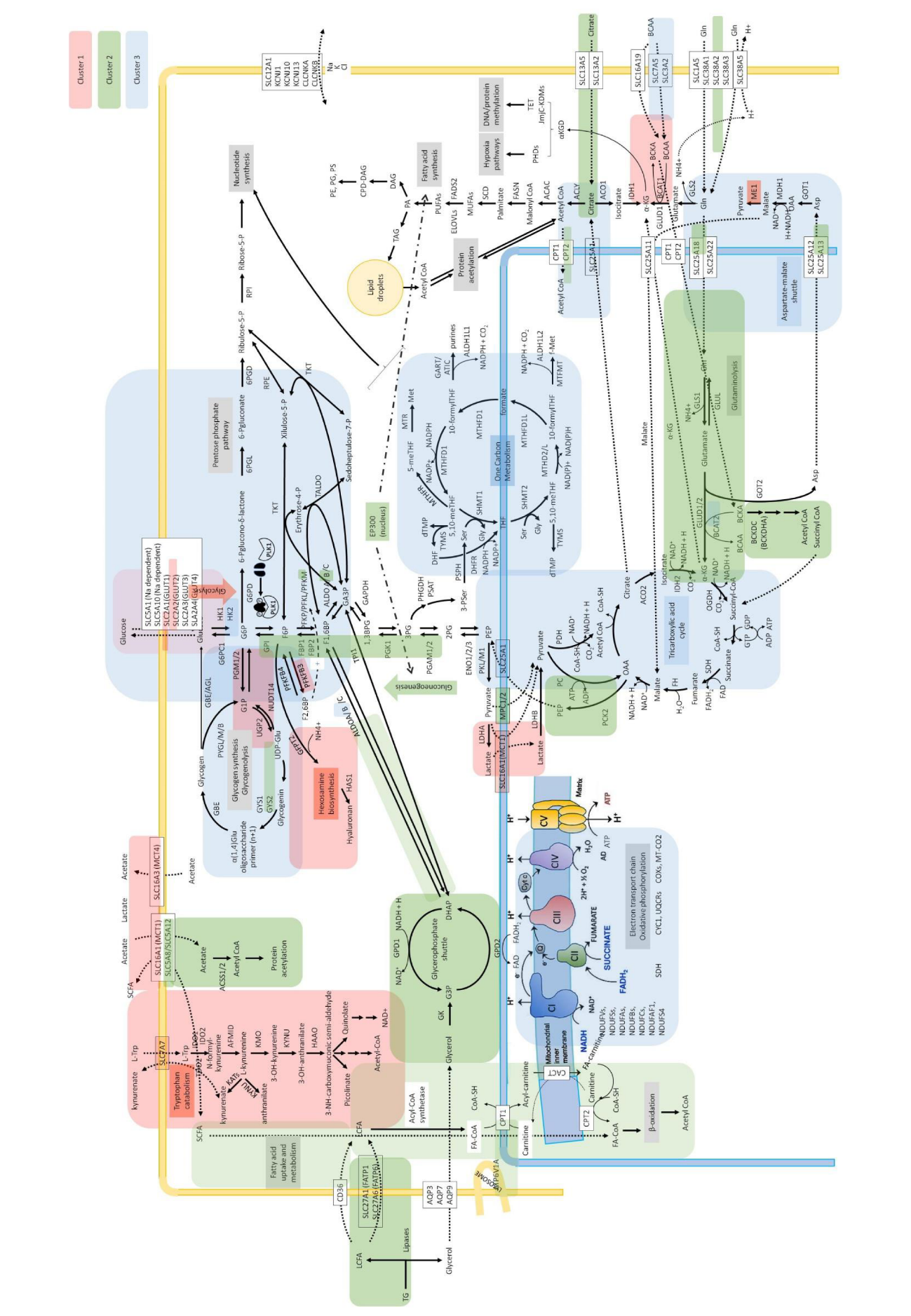


Supplementary Figure S4. Network integration metabolic pathways. Major activated pathways are highlighted in red in cluster 1, green in cluster 2 and in blue in cluster 3
