# Supplementary material for: A novel gene signature unveils three distinct immune-metabolic rewiring patterns conserved across diverse tumor types and associated with outcomes
Source: Front Immunol. 2022 Sep 2;13:926304. doi: 10.3389/fimmu.2022.926304 (PMC9479210; doi:10.3389/fimmu.2022.926304)
Supplement: Supplementary file 5 [file DataSheet_5.docx]

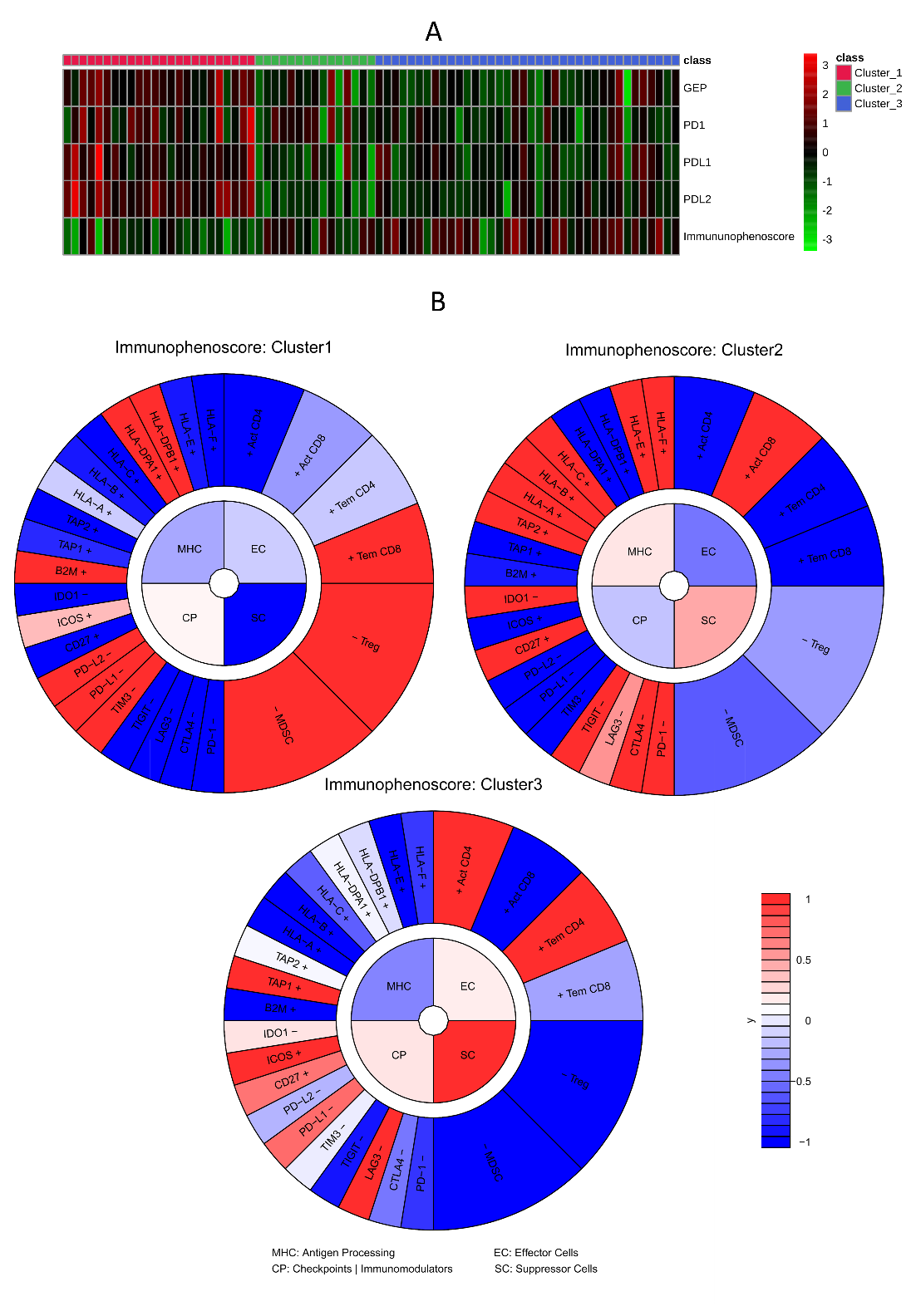


Supplementary Figure S5: Immune signatures and IMMETCOLS in 75 samples of 75 mCRC. A) heatmap of transcriptomics immune signatures in 75 mCRC stratified according to IMMETCOLS. GEP is the average expression of the genes of the GEP signature. Immunophenoscore is the aggregate of the MHC (Antigen Processing), EC (Effector cells), CP (Checkpoints and Immunomodulators) and SC (Suppressor cells) scores. B) Average immunophenogram in each IMMETCOLS cluster in the 75 mCRC. Inner circle plots each of the four Immunophenoscore components with higher values representing a more immunogenic phenotype.  The outer cycle plots the expression of markers used to compute each of the immunophenoscore components.
