# Supplementary material for: A novel gene signature unveils three distinct immune-metabolic rewiring patterns conserved across diverse tumor types and associated with outcomes
Source: Front Immunol. 2022 Sep 2;13:926304. doi: 10.3389/fimmu.2022.926304 (PMC9479210; doi:10.3389/fimmu.2022.926304)
Supplement: Supplementary file 6 [file DataSheet_6.docx]

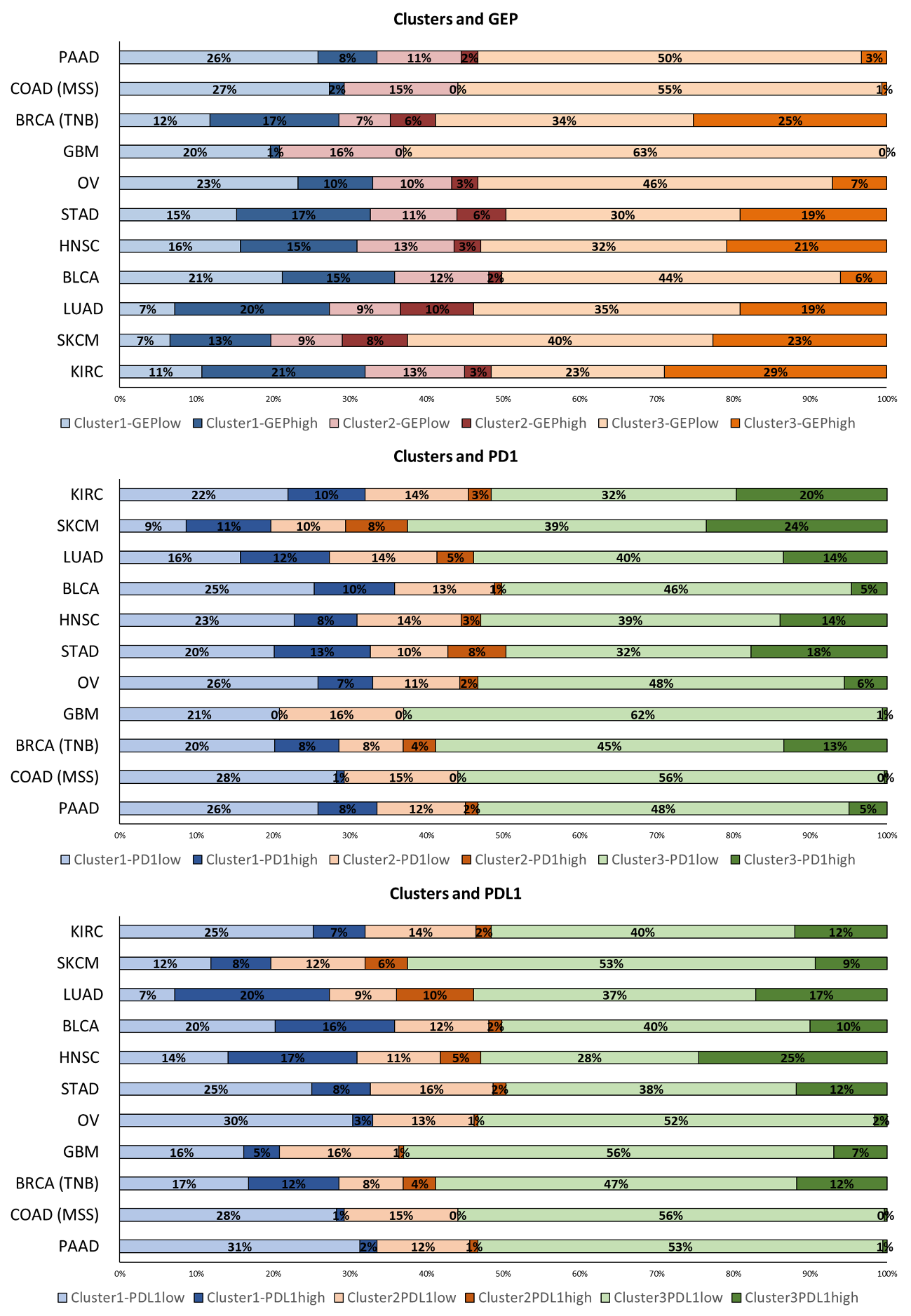


Supplementary Figure S6: Distribution of immune signatures and IMMETCOLS in each analyzed tumor type in TCGA. GEP high, PD1high, and PDL1high indicate the percentage of tumors above the 66th, 75th and 75th percentile for GEP, PD1 and PDL1 expression, respectively.
