# Supplementary material for: A novel gene signature unveils three distinct immune-metabolic rewiring patterns conserved across diverse tumor types and associated with outcomes
Source: Front Immunol. 2022 Sep 2;13:926304. doi: 10.3389/fimmu.2022.926304 (PMC9479210; doi:10.3389/fimmu.2022.926304)
Supplement: Supplementary file 8 [file Table_2.docx]

| **Signature** | **f.value** | **p.value** | **-LOG10(p)** | **FDR** | **Fisher's LSD** | | |
| --- | --- | --- | --- | --- | --- | --- | --- |
| **CAF** | 897.9 | 0 | Inf | 0 | Cluster_1 - Cluster_2 | Cluster_1 - Cluster_3 |  |
| **EMT** | 528.82 | 1,39E-202 | 205.86 | 9,71E-202 | Cluster_1 - Cluster_2 | Cluster_1 - Cluster_3 |  |
| **Hypoxia** | 217.08 | 1,20E-86 | 89.921 | 5,60E-86 | Cluster_1 - Cluster_2 | Cluster_1 - Cluster_3 | Cluster_3 - Cluster_2 |
| **Macropinocytosis** | 191.11 | 2,54E-77 | 79.595 | 8,89E-76 | Cluster_1 - Cluster_2 | Cluster_1 - Cluster_3 | Cluster_2 - Cluster_3 |
| **Fatty acid oxidation** | 69.3 | 2,33E-26 | 29.633 | 6,51E-26 | Cluster_2 - Cluster_1 | Cluster_3 - Cluster_1 | Cluster_2 - Cluster_3 |
| **Pentose phosphate pathway** | 61.572 | 4,23E-23 | 26.374 | 9,87E-23 | Cluster_2 - Cluster_1 | Cluster_3 - Cluster_1 | Cluster_3 - Cluster_2 |
| **Glycolysis** | 61.317 | 5,42E-23 | 26.266 | 1,08E-22 | Cluster_1 - Cluster_2 | Cluster_1 - Cluster_3 | Cluster_3 - Cluster_2 |
| **Cell cycle** | 36.317 | 2,27E-12 | 15.644 | 3,97E-12 | Cluster_1 - Cluster_2 | Cluster_3 - Cluster_1 | Cluster_3 - Cluster_2 |
| **Hexosamine biosynthesis** | 22.775 | 1,44E-06 | 98.402 | 2,25E-06 | Cluster_1 - Cluster_2 | Cluster_1 - Cluster_3 |  |
| **Glutamine metabolic process** | 22.473 | 1,95E-06 | 97.106 | 2,73E-06 | Cluster_2 - Cluster_1 | Cluster_3 - Cluster_1 | Cluster_2 - Cluster_3 |
| **Lactate transport** | 21.284 | 6,32E-06 | 9.199 | 8,05E-06 | Cluster_1 - Cluster_2 | Cluster_1 - Cluster_3 |  |
| **WNT pathway** | 13.417 | 1,55E-02 | 58.093 | 1,81E-02 | Cluster_1 - Cluster_2 | Cluster_1 - Cluster_3 |  |
| **Fatty acid biosyntesis** | 12.644 | 3,34E-02 | 54.757 | 3,60E-02 | Cluster_1 - Cluster_3 | Cluster_2 - Cluster_3 |  |
| **Regulation of autophagy** | 62.607 | 0.001927 | 27.151 | 0.001927 | Cluster_1 - Cluster_3 | Cluster_2 - Cluster_3 |  |

Supplementary Table S2: Important features identified by One-way ANOVA and post-hoc analysis (Fisher’s LSD) comparing the expression of different signatures between IMMETCOLS Clusters.
