# Supplementary material for: A novel gene signature unveils three distinct immune-metabolic rewiring patterns conserved across diverse tumor types and associated with outcomes
Source: Front Immunol. 2022 Sep 2;13:926304. doi: 10.3389/fimmu.2022.926304 (PMC9479210; doi:10.3389/fimmu.2022.926304)
Supplement: Supplementary file 9 [file Table_3.docx]

Supplementary Table S3: Important features identified by One-way ANOVA and post-hoc analysis (Fisher’s LSD) comparing the expression of metabolic genes in the IMMETCOLS Clusters. (Part 1)

| **Genes** | **f.value** | **p.value** | **(=)-LOG10(p)** | **FDR** | **Fisher's LSD** | | |
| --- | --- | --- | --- | --- | --- | --- | --- |
| **GFPT2** | 467.67 | 6,39E-181 | 184.19 | 6,01E-179 | Cluster_1 - Cluster_2 | Cluster_1 - Cluster_3 |  |
| **LDHA** | 303.37 | 3,62E-120 | 123.44 | 1,70E-118 | Cluster_1 - Cluster_2 | Cluster_3 - Cluster_2 |  |
| **HAS1** | 276.15 | 1,02E-109 | 112.99 | 3,19E-108 | Cluster_1 - Cluster_2 | Cluster_1 - Cluster_3 |  |
| **SLC2A3** | 267.62 | 2,02E-107 | 109.69 | 4,74E-105 | Cluster_1 - Cluster_2 | Cluster_1 - Cluster_3 | Cluster_3 - Cluster_2 |
| **BCAT1** | 219.99 | 8,55E-88 | 91.068 | 1,61E-86 | Cluster_1 - Cluster_2 | Cluster_1 - Cluster_3 | Cluster_2 - Cluster_3 |
| **GLUL** | 198.15 | 3,94E-79 | 82.405 | 6,17E-78 | Cluster_2 - Cluster_1 | Cluster_1 - Cluster_3 | Cluster_2 - Cluster_3 |
| **NDUFS3** | 192.02 | 1,10E-77 | 79.958 | 1,48E-75 | Cluster_2 - Cluster_1 | Cluster_3 - Cluster_1 | Cluster_3 - Cluster_2 |
| **NDUFV1** | 169.97 | 7,78E-68 | 71.109 | 9,14E-67 | Cluster_2 - Cluster_1 | Cluster_3 - Cluster_1 | Cluster_3 - Cluster_2 |
| **GOT1** | 159.76 | 1,03E-63 | 66.986 | 1,08E-62 | Cluster_2 - Cluster_1 | Cluster_3 - Cluster_1 | Cluster_3 - Cluster_2 |
| **NDUFA8** | 157.62 | 7,65E-63 | 66.116 | 7,19E-62 | Cluster_2 - Cluster_1 | Cluster_3 - Cluster_1 | Cluster_3 - Cluster_2 |
| **SLC7A7** | 148.23 | 4,98E-59 | 62.303 | 4,25E-58 | Cluster_1 - Cluster_2 | Cluster_1 - Cluster_3 | Cluster_2 - Cluster_3 |
| **CYC1** | 139.1 | 2,63E-56 | 58.581 | 2,06E-54 | Cluster_2 - Cluster_1 | Cluster_3 - Cluster_1 | Cluster_3 - Cluster_2 |
| **NDUFA9** | 124.83 | 1,84E-49 | 52.734 | 1,33E-48 | Cluster_2 - Cluster_1 | Cluster_3 - Cluster_1 | Cluster_3 - Cluster_2 |
| **GLS** | 124.52 | 2,47E-49 | 52.607 | 1,66E-48 | Cluster_2 - Cluster_1 | Cluster_1 - Cluster_3 | Cluster_2 - Cluster_3 |
| **SLC16A3** | 109.53 | 3,78E-43 | 46.422 | 2,37E-42 | Cluster_1 - Cluster_2 | Cluster_1 - Cluster_3 | Cluster_3 - Cluster_2 |
| **SLC25A1** | 107.96 | 1,68E-43 | 45.776 | 9,84E-42 | Cluster_2 - Cluster_1 | Cluster_3 - Cluster_1 | Cluster_3 - Cluster_2 |
| **CLCNKA** | 96.575 | 8,93E-38 | 41.049 | 4,94E-37 | Cluster_2 - Cluster_1 | Cluster_3 - Cluster_1 | Cluster_2 - Cluster_3 |
| **CPT2** | 96.159 | 1,33E-37 | 40.876 | 6,94E-37 | Cluster_2 - Cluster_1 | Cluster_3 - Cluster_1 |  |
| **PFKP** | 93.554 | 1,62E-36 | 39.791 | 8,00E-36 | Cluster_1 - Cluster_2 | Cluster_3 - Cluster_2 |  |
| **CCNB1** | 92.536 | 4,29E-36 | 39.367 | 2,02E-35 | Cluster_1 - Cluster_2 | Cluster_3 - Cluster_1 | Cluster_3 - Cluster_2 |
| **CLCNKB** | 80.472 | 4,72E-31 | 34.326 | 2,11E-30 | Cluster_2 - Cluster_1 | Cluster_2 - Cluster_3 |  |
| **SLC25A11** | 72.49 | 1,06E-27 | 30.975 | 4,52E-27 | Cluster_2 - Cluster_1 | Cluster_3 - Cluster_1 | Cluster_3 - Cluster_2 |
| **BCAT2** | 71.692 | 2,29E-27 | 30.64 | 9,36E-27 | Cluster_2 - Cluster_1 | Cluster_3 - Cluster_1 |  |
| **GLS2** | 69.704 | 1,57E-26 | 29.803 | 6,16E-27 | Cluster_2 - Cluster_1 | Cluster_3 - Cluster_1 | Cluster_2 - Cluster_3 |
| **SLC25A13** | 69.422 | 2,07E-26 | 29.685 | 7,77E-26 | Cluster_2 - Cluster_1 | Cluster_3 - Cluster_1 |  |
| **ACSS2** | 68.541 | 4,86E-26 | 29.314 | 1,76E-25 | Cluster_2 - Cluster_1 | Cluster_3 - Cluster_1 |  |
| **IDO1** | 65.276 | 1,16E-24 | 27.937 | 4,02E-24 | Cluster_1 - Cluster_2 | Cluster_1 - Cluster_3 | Cluster_3 - Cluster_2 |
| **PKM** | 63.834 | 4,69E-24 | 27.329 | 1,57E-23 | Cluster_1 - Cluster_2 | Cluster_3 - Cluster_1 | Cluster_3 - Cluster_2 |
| **CCNB2** | 62.111 | 2,50E-23 | 26.601 | 8,12E-23 | Cluster_1 - Cluster_2 | Cluster_3 - Cluster_1 | Cluster_3 - Cluster_2 |
| **TKT** | 61.242 | 5,83E-23 | 26.234 | 1,83E-22 | Cluster_2 - Cluster_1 | Cluster_3 - Cluster_1 | Cluster_3 - Cluster_2 |
| **NDUFV2** | 61.036 | 7,12E-23 | 26.147 | 2,16E-22 | Cluster_3 - Cluster_1 | Cluster_3 - Cluster_2 |  |
| **KCNJ1** | 60.045 | 1,87E-22 | 25.728 | 5,49E-23 | Cluster_2 - Cluster_1 | Cluster_1 - Cluster_3 | Cluster_2 - Cluster_3 |
| **SLC25A22** | 58.252 | 1,07E-21 | 24.97 | 3,05E-21 | Cluster_2 - Cluster_1 | Cluster_3 - Cluster_1 | Cluster_3 - Cluster_2 |
| **PFKL** | 58.046 | 1,31E-22 | 24.883 | 3,62E-21 | Cluster_2 - Cluster_1 | Cluster_3 - Cluster_1 | Cluster_3 - Cluster_2 |
| **KCNJ10** | 57.512 | 2,20E-21 | 24.657 | 5,92E-21 | Cluster_2 - Cluster_1 | Cluster_2 - Cluster_3 |  |
| **KCNJ13** | 54.921 | 2,76E-20 | 23.56 | 7,02E-20 | Cluster_2 - Cluster_1 | Cluster_3 - Cluster_1 | Cluster_2 - Cluster_3 |
| **PLK1** | 54.919 | 2,76E-20 | 23.559 | 7,02E-20 | Cluster_1 - Cluster_2 | Cluster_3 - Cluster_1 | Cluster_3 - Cluster_2 |
| **COX16** | 54.622 | 3,69E-20 | 23.433 | 9,13E-20 | Cluster_2 - Cluster_1 | Cluster_3 - Cluster_1 | Cluster_3 - Cluster_2 |
| **SLC3A2** | 54.183 | 5,67E-20 | 23.247 | 1,37E-19 | Cluster_2 - Cluster_1 | Cluster_3 - Cluster_1 | Cluster_3 - Cluster_2 |
| **PCK2** | 53.186 | 1,50E-19 | 22.824 | 3,52E-19 | Cluster_2 - Cluster_1 | Cluster_3 - Cluster_1 |  |
| **NDUFS4** | 52.381 | 3,29E-19 | 22.483 | 7,54E-19 | Cluster_1 - Cluster_2 | Cluster_3 - Cluster_1 | Cluster_3 - Cluster_2 |
| **PFKFB3** | 52.06 | 4,50E-19 | 22.346 | 1,01E-18 | Cluster_1 - Cluster_2 | Cluster_1 - Cluster_3 |  |
| **SLC16A1** | 50.647 | 1,79E-18 | 21.747 | 3,92E-18 | Cluster_1 - Cluster_2 | Cluster_1 - Cluster_3 | Cluster_3 - Cluster_2 |
| **CDK1** | 50.444 | 2,18E-19 | 21.661 | 4,67E-18 | Cluster_1 - Cluster_2 | Cluster_3 - Cluster_1 | Cluster_3 - Cluster_2 |
| **SLC12A1** | 50.405 | 2,27E-18 | 21.644 | 4,74E-18 | Cluster_2 - Cluster_1 | Cluster_2 - Cluster_3 |  |
| **MT.CO2** | 50.23 | 2,69E-18 | 21.57 | 5,50E-18 | Cluster_2 - Cluster_1 | Cluster_3 - Cluster_1 |  |

| **Genes** | **f.value** | **p.value** | **(=)-LOG10(p)** | **FDR** | **Fisher's LSD** | | |
| --- | --- | --- | --- | --- | --- | --- | --- |
| **CCNA2** | 50.198 | 2,78E-18 | 21.556 | 5,56E-18 | Cluster_1 - Cluster_2 | Cluster_3 - Cluster_1 | Cluster_3 - Cluster_2 |
| **UQCRFS1** | 50.119 | 3,00E-18 | 21.523 | 5,88E-18 | Cluster_2 - Cluster_1 | Cluster_3 - Cluster_1 | Cluster_3 - Cluster_2 |
| **SLC38A2** | 50.035 | 3,26E-18 | 21.487 | 6,25E-18 | Cluster_1 - Cluster_2 | Cluster_1 - Cluster_3 |  |
| **SLC13A2** | 48.34 | 1.71e-21 | 20.767 | 3,21E-17 | Cluster_2 - Cluster_1 | Cluster_3 - Cluster_1 | Cluster_2 - Cluster_3 |
| **SLC38A5** | 47.789 | 2,93E-17 | 20.533 | 5,40E-17 | Cluster_1 - Cluster_2 | Cluster_1 - Cluster_3 |  |
| **SDHA** | 45.883 | 1,90E-16 | 19.722 | 3,43E-16 | Cluster_3 - Cluster_1 | Cluster_3 - Cluster_2 |  |
| **HK2** | 43.852 | 1,39E-15 | 18.858 | 2,46E-16 | Cluster_1 - Cluster_2 | Cluster_3 - Cluster_2 |  |
| **EP300** | 43.351 | 2,27E-15 | 18.645 | 3,95E-15 | Cluster_2 - Cluster_1 | Cluster_1 - Cluster_3 | Cluster_2 - Cluster_3 |
| **IDH2** | 41.393 | 1,55E-14 | 17.81 | 2,65E-14 | Cluster_2 - Cluster_1 | Cluster_3 - Cluster_1 | Cluster_3 - Cluster_2 |
| **CDK2** | 41.003 | 2.27e-18 | 17.644 | 3,81E-14 | Cluster_3 - Cluster_1 | Cluster_3 - Cluster_2 |  |
| **PFKFB4** | 40.429 | 3,99E-15 | 17.399 | 6,58E-14 | Cluster_1 - Cluster_2 | Cluster_3 - Cluster_1 | Cluster_3 - Cluster_2 |
| **COX15** | 38.815 | 1,95E-13 | 16.71 | 3,16E-13 | Cluster_2 - Cluster_1 | Cluster_3 - Cluster_1 | Cluster_3 - Cluster_2 |
| **MPC1** | 38.714 | 2,15E-13 | 16.667 | 3,43E-13 | Cluster_2 - Cluster_1 | Cluster_3 - Cluster_1 |  |
| **SLC2A1** | 37.758 | 5,50E-13 | 16.259 | 8,62E-13 | Cluster_1 - Cluster_2 | Cluster_3 - Cluster_2 |  |
| **ATP6V1A** | 36.145 | 2,69E-13 | 15.57 | 4,14E-12 | Cluster_2 - Cluster_1 | Cluster_2 - Cluster_3 |  |
| **PC** | 31.227 | 3,42E-10 | 13.466 | 5,18E-10 | Cluster_2 - Cluster_1 | Cluster_3 - Cluster_1 |  |
| **BCKDK** | 30.434 | 7,47E-10 | 13.127 | 1,11E-09 | Cluster_1 - Cluster_2 | Cluster_3 - Cluster_1 | Cluster_3 - Cluster_2 |
| **CDK4** | 29.706 | 1,53E-09 | 12.815 | 2,25E-09 | Cluster_3 - Cluster_1 | Cluster_3 - Cluster_2 |  |
| **ME1** | 26.253 | 4,63E-08 | 11.334 | 6,70E-08 | Cluster_1 - Cluster_3 | Cluster_2 - Cluster_3 |  |
| **CCNE1** | 25.55 | 9,29E-08 | 11.032 | 1,32E-07 | Cluster_3 - Cluster_1 | Cluster_3 - Cluster_2 |  |
| **IDO2** | 22.251 | 2,43E-06 | 96.149 | 3,41E-06 | Cluster_1 - Cluster_3 | Cluster_2 - Cluster_3 |  |
| **SLC38A3** | 21.825 | 3,70E-06 | 94.319 | 5,11E-06 | Cluster_2 - Cluster_1 | Cluster_3 - Cluster_1 | Cluster_2 - Cluster_3 |
| **FBP2** | 21.303 | 6,20E-06 | 92.074 | 8,45E-06 | Cluster_2 - Cluster_1 | Cluster_2 - Cluster_3 |  |
| **GYS1** | 16.755 | 5,64E-04 | 7.249 | 7,57E-05 | Cluster_1 - Cluster_2 | Cluster_3 - Cluster_1 | Cluster_3 - Cluster_2 |
| **SLC38A1** | 16.465 | 7,52E-04 | 7.124 | 9.95e-08 | Cluster_1 - Cluster_2 | Cluster_1 - Cluster_3 |  |
| **PGM1** | 16.107 | 1,07E-03 | 69.699 | 1,40E-03 | Cluster_1 - Cluster_2 | Cluster_3 - Cluster_2 |  |
| **HK1** | 15.384 | 2,20E-03 | 66.581 | 2,83E-03 | Cluster_1 - Cluster_2 | Cluster_1 - Cluster_3 |  |
| **PFKM** | 15.271 | 2,46E-03 | 66.091 | 3,12E-03 | Cluster_2 - Cluster_1 | Cluster_3 - Cluster_1 |  |
| **BCKDHA** | 13.966 | 8,99E-03 | 60.463 | 1,13E-02 | Cluster_2 - Cluster_1 | Cluster_3 - Cluster_1 | Cluster_2 - Cluster_3 |
| **FBP1** | 13.597 | 1,30E-02 | 58.871 | 1,60E-02 | Cluster_2 - Cluster_1 | Cluster_2 - Cluster_3 |  |
| **CCNE2** | 13.357 | 1,65E-02 | 57.832 | 2,01E-02 | Cluster_1 - Cluster_2 | Cluster_3 - Cluster_1 | Cluster_3 - Cluster_2 |
| **SLC7A5** | 12.986 | 2,38E-02 | 5.623 | 2,87E-02 | Cluster_3 - Cluster_1 | Cluster_3 - Cluster_2 |  |
| **G6PD** | 12.856 | 2,71E-02 | 55.671 | 3,22E-02 | Cluster_3 - Cluster_1 | Cluster_3 - Cluster_2 |  |
| **GK** | 95.348 | 7,38E-01 | 4.132 | 8,67E-02 | Cluster_2 - Cluster_1 | Cluster_3 - Cluster_1 | Cluster_2 - Cluster_3 |
| **GLUD2** | 93.513 | 8,86E-01 | 40.526 | 0.00010281 | Cluster_2 - Cluster_1 | Cluster_2 - Cluster_3 |  |
| **GYS2** | 8.419 | 0.00022421 | 36.493 | 0.00025702 | Cluster_2 - Cluster_1 | Cluster_3 - Cluster_1 |  |
| **SLC1A5** | 76.397 | 0.00048738 | 33.121 | 0.00055197 | Cluster_3 - Cluster_1 | Cluster_3 - Cluster_2 |  |
| **SLC5A12** | 74.401 | 0.00059463 | 32.258 | 0.00066542 | Cluster_2 - Cluster_1 | Cluster_2 - Cluster_3 |  |
| **SLC13A5** | 73.449 | 0.0006538 | 31.846 | 0.00072303 | Cluster_1 - Cluster_3 | Cluster_2 - Cluster_3 |  |
| **SLC27A6** | 72.406 | 0.00072544 | 31.394 | 0.00079293 | Cluster_1 - Cluster_3 | Cluster_2 - Cluster_3 |  |
| **SLC25A18** | 72.219 | 0.00073907 | 31.313 | 0.00079854 | Cluster_2 - Cluster_1 | Cluster_3 - Cluster_1 |  |
| **PGM2** | 7.003 | 0.00091927 | 30.366 | 0.00098194 | Cluster_1 - Cluster_2 | Cluster_1 - Cluster_3 |  |
| **ALDOB** | 65.517 | 0.0014417 | 28.411 | 0.0015227 | Cluster_2 - Cluster_1 | Cluster_2 - Cluster_3 |  |
| **SLC5A8** | 63.515 | 0.0017602 | 27.544 | 0.0018384 | Cluster_2 - Cluster_1 | Cluster_3 - Cluster_1 |  |
| **BRCA2** | 62.289 | 0.001989 | 27.014 | 0.0020545 | Cluster_1 - Cluster_2 | Cluster_3 - Cluster_2 |  |
| **GPD1** | 42.504 | 0.014317 | 18.441 | 0.014628 | Cluster_2 - Cluster_1 | Cluster_2 - Cluster_3 |  |
| **SLC5A1** | 35.952 | 0.027534 | 15.601 | 0.027831 | Cluster_3 - Cluster_1 | Cluster_3 - Cluster_2 |  |
| **SLC5A10** | 31.935 | 0.041125 | 13.859 | 0.041125 | Cluster_3 - Cluster_1 |  |  |

Supplementary Table S3: Important features identified by One-way ANOVA and post-hoc analysis (Fisher’s LSD) comparing the expression of metabolic genes in the IMMETCOLS Clusters. (Part 2)
