# Supplementary material for: A novel gene signature unveils three distinct immune-metabolic rewiring patterns conserved across diverse tumor types and associated with outcomes
Source: Front Immunol. 2022 Sep 2;13:926304. doi: 10.3389/fimmu.2022.926304 (PMC9479210; doi:10.3389/fimmu.2022.926304)
Supplement: Supplementary file 10 [file Table_4.docx]

Supplementary Table S4: Important features identified by One-way ANOVA and post-hoc analysis (Fisher’s LSD) comparing the expression of immune signatures in the IMMETCOLS Clusters.

| **Signatures** | **f.value** | **p.value** | **(-)LOG10(p)** | **FDR** | **Fisher's LSD** | | |
| --- | --- | --- | --- | --- | --- | --- | --- |
| **Macrophages** | 367.84 | 1,81E-144 | 147.74 | 2,35E-143 | Cluster_1 - Cluster_2 | Cluster_1 - Cluster_3 | Cluster_2 - Cluster_3 |
| **CD45** | 286.94 | 7,14E-114 | 117.15 | 4,64E-113 | Cluster_1 - Cluster_2 | Cluster_1 - Cluster_3 | Cluster_2 - Cluster_3 |
| **NK cells** | 146.54 | 2,43E-58 | 61.615 | 1,05E-57 | Cluster_1 - Cluster_2 | Cluster_1 - Cluster_3 | Cluster_2 - Cluster_3 |
| **Neutrophils** | 124.63 | 2,23E-50 | 52.653 | 7,23E-49 | Cluster_1 - Cluster_2 | Cluster_1 - Cluster_3 | Cluster_2 - Cluster_3 |
| **T cells** | 122.97 | 1,08E-48 | 51.968 | 2,80E-48 | Cluster_1 - Cluster_2 | Cluster_1 - Cluster_3 |  |
| **T reg** | 121.82 | 3,20E-48 | 51.495 | 6,93E-48 | Cluster_1 - Cluster_2 | Cluster_1 - Cluster_3 |  |
| **Th1 cells** | 112.89 | 1,53E-44 | 47.815 | 2,85E-44 | Cluster_1 - Cluster_2 | Cluster_1 - Cluster_3 |  |
| **CD8-T cells** | 92.408 | 4,85E-36 | 39.314 | 7,88E-36 | Cluster_1 - Cluster_2 | Cluster_1 - Cluster_3 |  |
| **Th2 cells** | 86.21 | 1,88E-33 | 36.727 | 2,71E-34 | Cluster_1 - Cluster_2 | Cluster_3 - Cluster_1 | Cluster_3 - Cluster_2 |
| **DC** | 67.49 | 1,35E-25 | 28.871 | 1,75E-25 | Cluster_1 - Cluster_2 | Cluster_1 - Cluster_3 |  |
| **Bcells** | 47.568 | 3,64E-17 | 20.439 | 4,30E-17 | Cluster_1 - Cluster_2 | Cluster_1 - Cluster_3 | Cluster_2 - Cluster_3 |
| **Cytotoxic cells** | 25.127 | 1,41E-07 | 10.851 | 1,53E-07 | Cluster_1 - Cluster_3 | Cluster_2 - Cluster_3 |  |
| **T helper cells** | 43.796 | 0.012584 | 19.002 | 0.012584 | Cluster_1 - Cluster_3 | Cluster_2 - Cluster_3 |  |
