# Supplementary material for: A novel gene signature unveils three distinct immune-metabolic rewiring patterns conserved across diverse tumor types and associated with outcomes
Source: Front Immunol. 2022 Sep 2;13:926304. doi: 10.3389/fimmu.2022.926304 (PMC9479210; doi:10.3389/fimmu.2022.926304)

Supplementary Table S5: Important features identified by One-way ANOVA and post-hoc analysis (Fisher’s LSD) comparing the expression of immune genes in the IMMETCOLS Clusters.


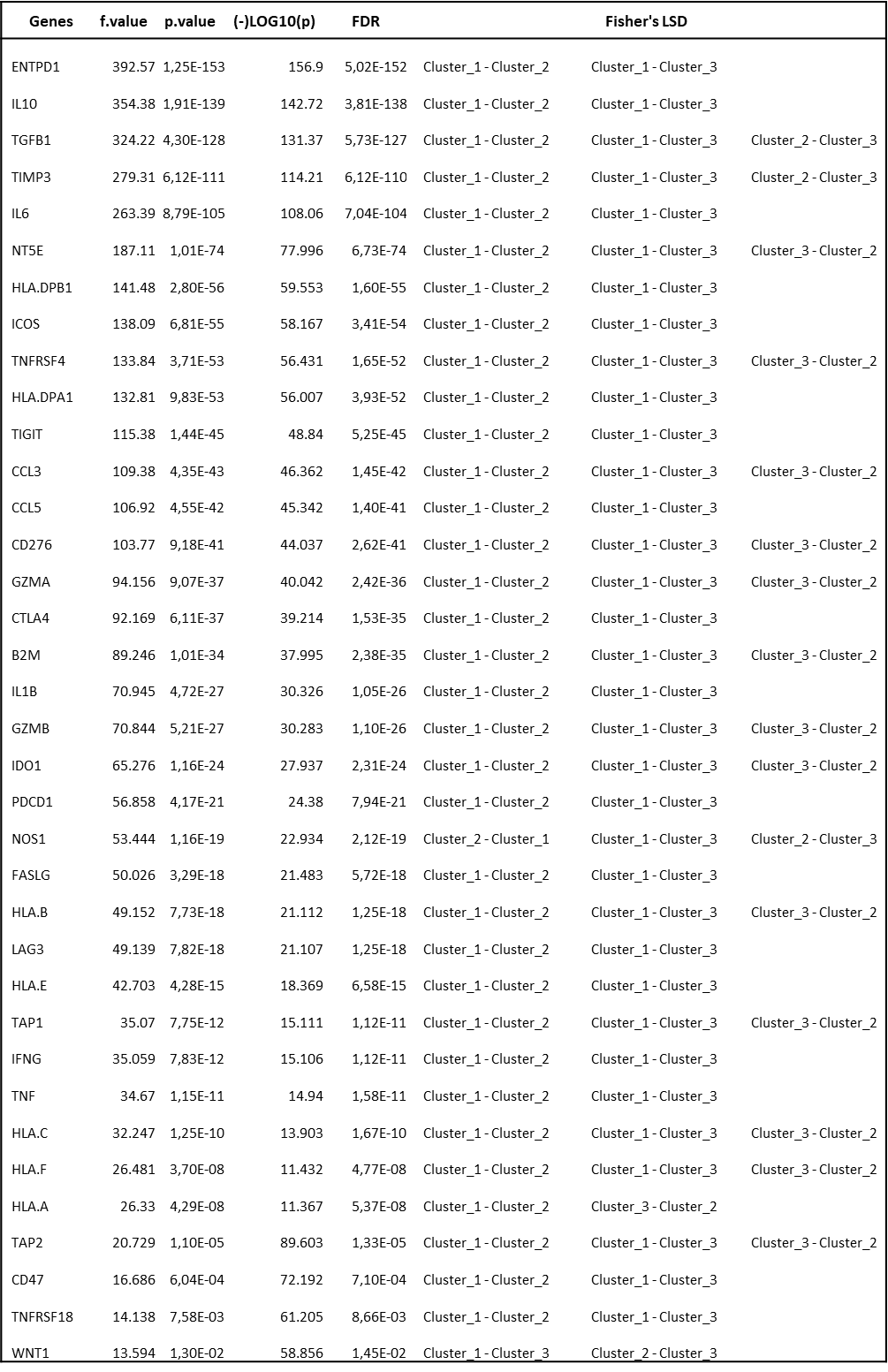

Supplement: Supplementary file 11 [file Table_5.docx]
